# Applying Rasch analysis to evaluate measurement equivalence of different administration formats of the Activity Limitation scale of the Cambridge Pulmonary Hypertension Outcome Review (CAMPHOR)

**DOI:** 10.1186/s12955-016-0462-2

**Published:** 2016-04-09

**Authors:** J. Twiss, S. P. McKenna, J. Graham, K. Swetz, J. Sloan, M. Gomberg-Maitland

**Affiliations:** Galen Research, B1 Chorlton Mill, 3 Cambridge Street, Manchester, M1 5BY UK; Mayo Clinic, Rochester, Minnesota USA; University of Chicago Medical Center, Chicago, Illinois USA

**Keywords:** Patient reported outcome (PRO) measures, Cambridge Pulmonary Hypertension Outcome Review (CAMPHOR), Item response theory (IRT), Rasch analysis, Electronic validation, Measurement equivalence

## Abstract

**Background:**

Electronic formats of patient-reported outcome (PRO) measures are now routinely used in clinical research studies. When changing from a validated paper and pen to electronic administration it is necessary to establish their equivalence. This study reports on the value of Rasch analysis in this process.

**Methods:**

Three groups of US pulmonary hypertension (PH) patients participated. The first completed an electronic version of the CAMPHOR Activity Limitation scale (e-sample) and this was compared with two pen and paper administrated samples (pp1 and pp2). The three databases were combined and analysed for fit to the Rasch model. Equivalence was evaluated by differential item functioning (DIF) analyses.

**Results:**

The three datasets were matched randomly in terms of sample size (*n* = 147). Mean age (years) and percentage of male respondents were as follows: e-sample (51.7, 16.0 %); pp1 (50.0, 14.0 %); pp2 (55.5, 40.4 %). The combined dataset achieved fit to the Rasch model. Two items showed evidence of borderline DIF. Further analyses showed the inclusion of these items had little impact on Rasch estimates indicating the DIF identified was unimportant.

**Conclusions:**

Differences between the performance of the electronic and pen and paper administrations of the CAMPHOR Activity Limitation scale were minor. The results were successful in showing how the Rasch model can be used to determine the equivalence of alternative formats of PRO measures.

## Background

Patient reported outcomes (PROs) provide a crucial means of assessing the impact of a condition and its treatment from the patients’ perspective. It has become increasingly common for PROs to be used as secondary (and even primary) endpoints in clinical trials [1]. In such studies electronic formats are generally employed. These may offer a range of benefits such as improving access to patients, increasing compliance, reducing missing data and avoiding errors associated with manual data entry [2–4]. Adapting pen and paper measures for use electronically requires a number of format changes. These may include changes to item presentation, instructions and/or response format. Various electronic devices are used including personal computers, tablets, cell phones and handheld devices. Smaller devices often entail major formatting changes as they require the PRO to be broken into small sections to fit the screen. The way in which participants are required to respond on these devices can differ; for example, by using a standard mouse or a touch screen monitor. An important question is whether or not changes in format affect data generated from the measure. In order to investigate this it is necessary for the new format to undergo formal equivalence testing.

A recent literature review showed that several studies have attempted to assess equivalence between different formats of PROs [5]. However, the way in which equivalence is tested varies from study to study. These methods usually involve comparing sample means and/or assessing the level of agreement between the different formats using correlational techniques. Such studies have design limitations and only assess a small aspect of true ‘measurement equivalence’. For example, the statistic most widely used for assessing association is the intraclass correlation coefficient (ICC). However, the ICC requires interval/ratio level data, making its use inappropriate with most PROs currently available that produce data at the ordinal level [6].

Studies of association usually use a cross-over design in which participants are randomized to complete either a paper and pen version or an electronic version of the same PRO and then the other mode on a second administration. Generally, the time between administrations is short, with patients completing both formats at the same visit [7–9]. This is likely to lead to recall bias and inflated levels of agreement.

An alternative design employed is a parallel groups design in which participants are randomized to complete either a paper and pen version or an electronic version of the PRO. Mean scores on the measures are then compared and differences in these either considered in relation to minimal important difference values (if available) or effect sizes. Unfortunately, this approach gives markedly less information about measurement equivalence and is liable to bias if the samples are not closely matched on clinical characteristics.

A guidance document produced by the International Society for Pharmacoeconomics and Outcomes Research ePRO Good Research Practices Task Force [10] provides recommendations on the steps necessary to establish equivalence. The document suggests that respondent usability should always be assessed and that measurement equivalence should be tested quantitatively when ‘moderate’ changes are made to the format. The document follows common practice by recommending use of one of the two designs described above.

One of the major advances in PRO development and application in recent years has been the adoption of Item Response Theory (IRT) and, in particular, Rasch analysis [11]. Unlike Classical Test Theory (CTT), IRT gives greater emphasis to item-level rather than test-level information and provides superior detail on the measurement properties of scales [12–14]. Although IRT has been identified as a potentially useful method for evaluating measurement equivalence [10] a review of the literature identified just two studies applying this approach [15, 16].

The Rasch model allows a series of detailed properties to be evaluated that can be applied to the assessment of measurement equivalence. These include individual item fit statistics, overall fit statistics, item severity ordering and the functioning of response options. In addition, it allows measurement bias at the item level to be assessed using differential item functioning (DIF). This is of particular relevance to measurement equivalence as DIF by mode of administration (paper and pen versus electronic) can be tested.

The aim of the present study was to compare the measurement equivalence of pen and paper and electronic formats of the Activity Limitation scale of the Cambridge Pulmonary Hypertension Outcome Review (CAMPHOR) [17] using Rasch analysis.

## Methods

### CAMPHOR Activity Limitation

The CAMPHOR Activity Limitation scale employs the World Health Organization’s Classification of Functioning, Disability and Health [18] and consists of 15 items with three response options (Able to do on own without difficulty/Able to do on own with difficulty/Unable to do on own). Rasch analysis was used in the development of this scale to ensure unidimensionality and good measurement properties.

An electronic version of the scale was designed to resemble the original paper and pen version as closely as possible. The items were presented on whole pages in the same way as the original. A minor change to the response format was made with participants asked to ‘click’ the relevant response option using the cursor. In addition, the instructions were changed with patients asked to ‘click’ their response rather than to ‘check a box’. All other instructions were identical to the paper and pen version.

### Samples

Three separate samples of PH patients were included in the study. One sample completed an electronic version of the CAMPHOR and the other samples filled in a paper and pen version. All samples consisted of US patients with PH. Subscribers to listservs of the Pulmonary Hypertension Association, recruited by the Mayo Clinic, Rochester, completed an electronic version of the CAMPHOR (*e-sample*) [19]. A second sample, drawn from the Adelphi Real World Pulmonary Arterial Hypertension Disease Specific Programme [20], completed the paper and pen CAMPHOR at outpatient clinics and specialist PH centres (*pp1-sample*). The second paper and pen sample (*pp2-sample*) was recruited by the University of Chicago from a tertiary referral centre [21]. The original *e-sample* and *pp1* samples consisted of 276 and 151 participants respectively. Random samples (generated via SPSS 19 random sample selection generator) of 147 patients were chosen from each of these clinical samples to match the size of the *pp2* sample.

For the *e-sample,* ethics approval was provided by the Mayo Clinic Institutional Review Board. The *pp-1 sample* dataset was collected through a market research survey and accordingly ethics approval was not sought. For the *pp-2 sample,* ethics approval was provided by the University of Chicago Institutional Review Board. Informed consent was given by participants in all studies.

The samples were not matched for disease severity and the CAMPHOR was administered in different locations (clinic or home).

### Design

Data generated from the three samples of PH patients were compared. The design allowed any lack of equivalence between the functioning of electronic and pen and paper formats to be considered in the context of two different pen and paper administrations.

**Fig. 1:**
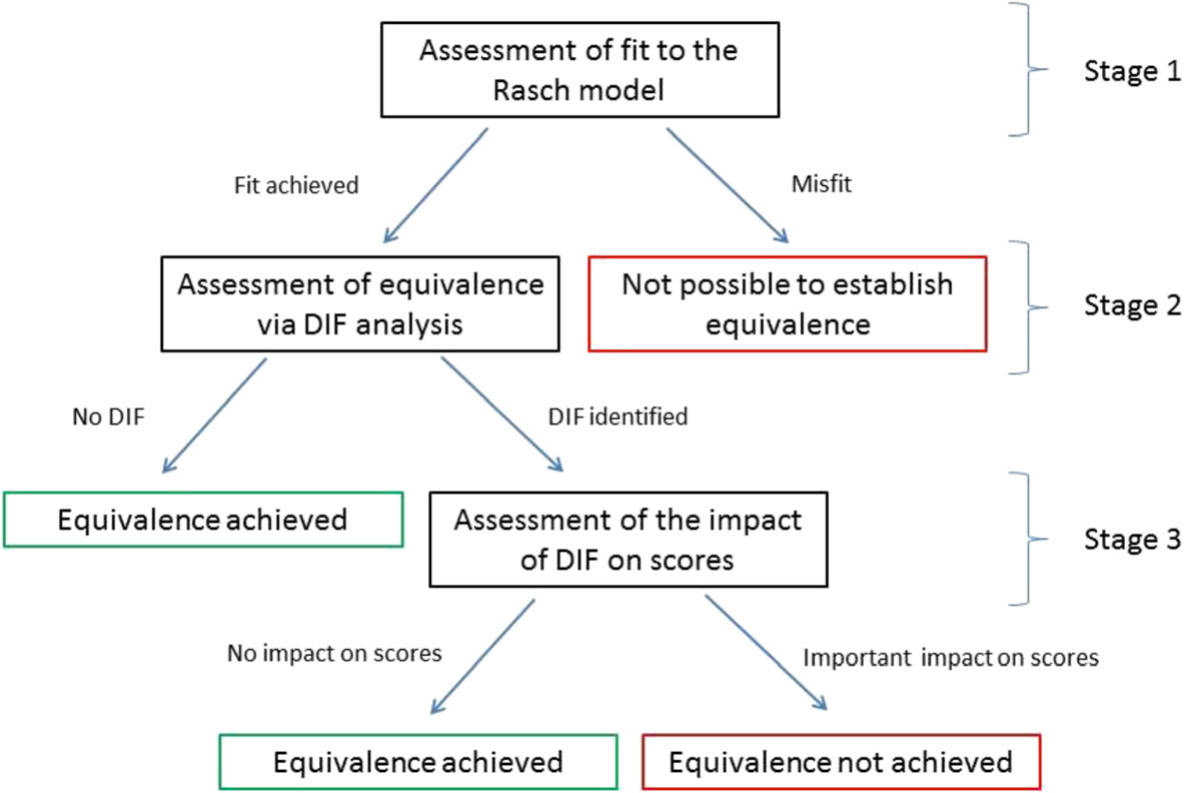
Design of the study

Figure 1 provides a flow chart detailing the design of the study. The three datasets were combined for the analyses. Three stages were involved:

1. Assessment of fit to the Rasch model: In order to assess equivalence, it was first necessary to re-establish that the combined scales were unidimensional with good fit to the Rasch model. Therefore, the analyses assessed the PRO scales’ standard fit statistics [22]. If fit statistics are adequate it is possible to assess measurement equivalence.
2. Measurement equivalence was assessed by investigating DIF [23] by method of administration. DIF occurs when sub-groups of a sample respond in different ways to a particular item, despite having the same level of the underlying trait being measured. This provides a way of assessing whether different formats of a measure produce bias at the item level. If there is no evidence of DIF then the measure exhibits equivalence. If DIF is shown it is necessary to assess its importance. If it is non-significant then equivalence is assumed.

The analyses are described in detail below.

1. ***Fit to the Rasch model***
  *The Rasch model*
  The Rasch model is a simple logistic latent trait IRT model [11]. Based on a probabilistic form of Guttman scaling, it states that items and persons can be located on the same linear scale. The probability of a given respondent affirming an item is defined as the relative distance between the item location (difficulty) and the respondent location (ability) on that scale. In relation to health outcome, it is a logistic function of the difference between a person’s severity level and the severity level of the item. In order to assess the measurement properties of a questionnaire formally, the response pattern created by the items is compared to that expected by the Rasch model. If the observed data do not deviate significantly from the expected responses the items fit the Rasch model.
  The Rasch Unidimensional Measurement Model (RUMM) 2030 [24] program was employed.
  Before analyses were conducted it was necessary to determine whether the rating scale [25] or partial credit model [26] was most appropriate. Both of these approaches use the Rasch model but differ slightly in their mathematics. The rating scale model is more stringent in its requirements as the distance between response thresholds has to be uniform across all items. The likelihood ratio test is used to identify the most appropriate model.
  *Overall fit to the Rasch model*
  Fit to the Rasch model was examined by looking at the overall item-trait interaction Chi^2^ fit value. A non-significant Chi^2^ statistic (*p* > 0.05) indicates that the scale fits the Rasch model. Bonferroni corrections were applied to the statistical p-values throughout the analyses due to the large number of tests conducted (Bonferroni adjusted *p value* > 0.003).
  *Individual item fit*
  Individual item fit statistics were also used to investigate overall fit to the Rasch model. A significant Chi^2^ fit statistic (*p* < 0.05 (Bonferroni adjusted)) indicates misfit to the model. Fit residuals also provide a valuable source of information. These should fall within ±2.5. High negative residuals suggest that an item may be redundant, whereas high positive residuals suggest multi-dimensionality. In both cases they are indicative of misfit. Person fit residual and item fit residual statistics are transformed by RUMM to approximate z-scores. These z-scores, the residual mean values of person and item fit, can also be used to assess overall fit to the Rasch model. Perfect fit is represented by a mean anchored at 0 and a standard deviation of 1.
  *Internal Reliability – Person Separation Index (PSI)*
  The Person Separation Index (PSI) was employed to analyse internal reliability. This indicates the power of the items to distinguish between respondents. A PSI score of 0.8 is considered the minimum acceptable level.
  *Functioning of response options*
  Response thresholds are investigated to determine the adequacy of the response format. Thresholds represent locations on the latent continuum at which it will be equally likely that adjacent response options will be selected. Thresholds should be logically ordered. If response options are disordered they do not work as intended. This is an important area for the present study as the method of responding could influence the thresholds.
  *Unidimensionality*
  As noted above, evidence of unidimensionality is first investigated by looking at the item fit residuals. Evidence of unidimensionality was also investigated using t-tests to compare estimates derived from two separate subsets of items (loading most differently on the first factor of the Residual Principal Component Analysis) [27]. If a scale is unidimensional these two subsets of items should give the same estimate of person ability. Generally, if more than 5 % of these t-tests are statistically significantly different, it is indicative of a multidimensional scale. A 95 % binomial confidence interval (using a normal approximation) is provided to indicate the interval within which the true value lies in repeated testing.
2. ***Assessment of equivalence***
  DIF was employed to test for equivalence between the different formats of the Activity Limitation scale. Analyses of variance (ANOVA) were employed to identify DIF by administration mode (*e-sample* versus *pp1* versus *pp2*). Significant p-values (*p* < 0.05) indicate the presence of DIF.
3. ***Assessing the impact of DIF***
  If DIF is identified it is necessary to assess the extent to which it influences the calculation of the Rasch estimates. If DIF is minor, its influence on estimates may be only slight. The importance of any identified DIF was tested using a method outlined by Tenant and Pallant [28]. Rasch estimates are first calculated using a ‘pure’ dataset where items exhibiting DIF are removed. These estimates are then saved to an anchor file. The whole dataset, including items exhibiting DIF, are anchored to this dataset so that the estimates are defined by the measurement framework of the ‘pure’ items. The resulting estimates (pure vs. full anchored dataset) are then compared. The proportion of estimates that differ by 0.5 logits is calculated to assess for the proportion of non-trivial DIF. Distribution of the estimates (including mean, standard deviation, median and interquartile range), correlation (intraclass correlation coefficient) between estimates and differences between the estimates (t-test) were also calculated.

## Results

Demographic information for the three samples is shown in Table 1. World Health Organization functional (WHO) classification and Six Minute Walk Test (6MWT) scores at time of administration of the CAMPHOR were only available for the two paper and pen samples. The *pp1* sample had a longer duration of PH while the *pp2* sample included a higher proportion of males. The WHO classifications suggested that the *pp1* sample had greater disease severity which was supported by the CAMPHOR Activity Limitation raw scores. The e-*sample* had comparable Activity Limitation scores to the *pp1* sample.

**Table 1:** Sample characteristics

	e-sample (*n* = 147)	*pp*1 (*n* = 147)	*pp*2 (*n* = 147)
Gender (%)			
Male	23 (16)	20 (14)	57 (40.4)
Female	121 (84)	123 (84)	84 (59.6)
Age (years)			
Mean (SD)	51.7 (13.9)	50.0 (14.6)	55.5 (12.7)
Range	20.0–82.0	16.0–82.0	21.0–93.0
PH duration (years)			
Mean (SD)	4.7 (3.8)	12.1 (4.9)	4.3 (4.0)
Range	0.0–19.0	7.0–29.0	3.0–21.0
6MWT (metres)			
Mean (SD)	-	385.6 (112.6)	396.7 (170.1)
Range	-	155.0–701.0	55.0–862.0
WHO classification (%)			
I	-	4 (2.9)	132 (95)
II	-	58 (42.3)	2 (1.4)
III	-	73 (53.3)	4 (2.9)
IV	-	2 (1.5)	0 (0)
V	-	0 (0)	1 (0.7)
PH type (%)			
Idiopathic PH/Familial PH	68 (46.3)	81 (55.1)	67 (45.6)
Associated PH	17 (11.6)	62 (42.2)	65 (44.2)
PH due to left heart disease	9 (6.1)	0 (0)	2 (1.4)
PH due to lung conditions	10 (6.8)	0(0)	4 (2.9)
PH due to other conditions	24 (16.3)	(0)	1 (0.7)
Reasons for PH unknown	19 (12.9)	4 (2.7)	8 (5.4)
CAMPHOR Activity limitation raw score			
Mean (SD)	18.07 (3.99)	17.91 (3.11)	8.04 (6.33)
Median (IQR)	18.0 (15.0–21.0)	18.0 (16.0–20.0)	7.0 (3.0–13.0)
%min	0.7	0.7	17.7
%max	0.7	2.0	0.7
Range	4.0–30.0	6.0–26.0	0.0–26.0

Further investigation was conducted using DIF analysis to assess whether differences in severity could have impacted on the equivalence testing results. There was no evidence of DIF by WHO classification (*pp1* vs. *pp2*) after Bonferroni corrections were applied.

### Fit to the Rasch model

The initial Likelihood Ratio test, used to determine the most appropriate Rasch model, was statistically significant supporting the use of the partial credit model for the analyses (*p* < .001).

Table 2 shows overall fit statistics for the combined samples. The combined dataset showed fit to the Rasch model (Chi^2^ = 0.028). In addition, the person separation index statistics indicated that the scale showed good reliability. Individual item fit is shown in Table 3. Each of the 15 items demonstrated fit to the Rasch model after Bonferroni corrections were applied (Chi^2^ ˃ 0.05).

**Table 2:** Overall fit statistics for the combined dataset

Scale	Item-trait interaction	PSI	Item-person fit residuals				Unidimensionality (95 % Confidence Interval)
	Chi^2^ *P*-value		Item fit residual		Person fit residual		
			Mean	SD	Mean	SD	
Activity limitation	0.027	0.907	−0.77	0.99	−0.45	1.02	0.064 (0.042–0.085)
Ideal values	>0.003^a^	>0.8	0	± 1	0	± 1	<0.05

^a^Bonferroni adjusted

**Table 3:** Individual Item Fit

Item	Location	SE	Fit residual	Chi^2^	Prob
Cut toenails	0.25	0.11	0.81	14.61	0.024
Bathe	2.02	0.14	−1.32	5.60	0.470
Get dressed	2.64	0.15	−0.35	7.02	0.319
Walk around house	2.52	0.15	−1.14	5.87	0.437
Walk short level distances	1.82	0.14	−1.43	4.49	0.611
Walk longer level distance	−0.37	0.11	−1.87	5.65	0.464
Walk up incline	−0.30	0.12	−2.15	10.39	0.109
Climb a flight of stairs	−1.18	0.11	−1.89	9.48	0.148
Bend to pick up objects	0.80	0.12	0.12	7.46	0.280
Stand for short time	2.20	0.15	0.51	4.23	0.646
Stand for long time	−0.76	0.11	−0.43	4.70	0.583
Lift heavy objects	−2.77	0.10	0.43	15.67	0.016
Carry heavy items	−3.42	0.11	−0.07	3.95	0.684
Do light house work	−0.10	0.11	−2.04	10.30	0.113
Do heavy housework	−3.33	0.11	−0.73	8.07	0.233

**Fig. 2:**
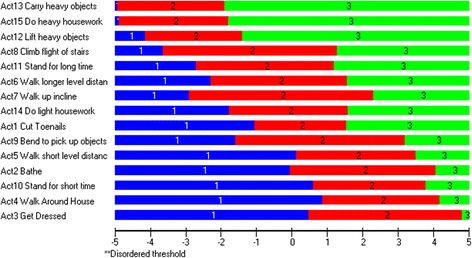
Response threshold map

Response thresholds for each item (in location order) are shown in Fig. 2. The results showed that the response format worked appropriately for all items. No disordered response thresholds were identified.

### Assessment of equivalence

None of the items showed DIF by administration after application of Bonferroni corrections. However, one item (item 5, *p* = 0.003) showed borderline non-uniform DIF and another showed borderline uniform DIF (item 12, *p* = 0.002). These items were investigated in more detail.

**Fig. 3:**
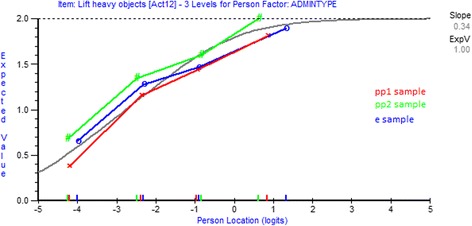
DIF by administration for item 12 (uniform)

Figure 3 shows uniform DIF by administration for item 12 (Lift heavy objects). The curved line represents expected scores by class of people or class of interval for this item and the points represent the observed scores for each of the three samples. Members of the *pp2* sample were most likely to affirm the item at each point. A post-hoc analysis supported this, showing that the *pp2* sample differed significantly from the *pp1* sample and the *e-sample*.

**Fig. 4:**
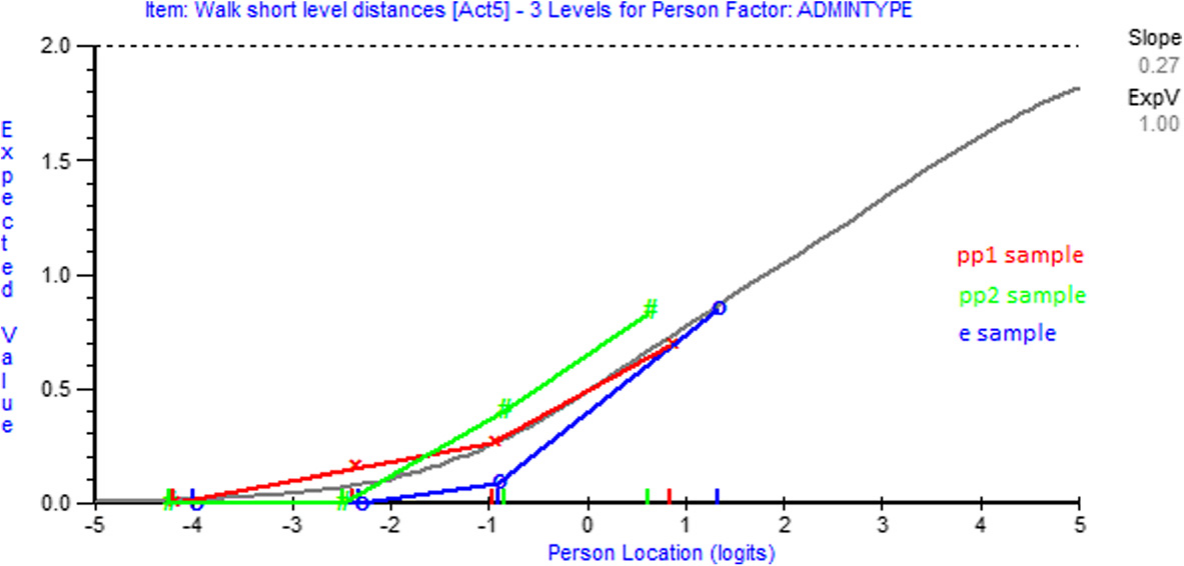
DIF by administration for item 5 (non-uniform)

Figure 4 shows non-uniform DIF by administration for item 5 (Walk short distances on level ground). As this DIF was non-uniform, it was not possible to identify an administration mode that differed consistently from the others.

In order to assess the level of bias exhibited by items 5 and 12, Rasch estimates were compared between a pure dataset excluding these items and an anchored dataset including them. There were only minor differences between the two estimates (with DIF items mean (SD) = −2.17 (2.38), without DIF items mean (SD) = −2.19 (2.41), t-test *p* = 0.224). Only 3.4 % of person estimates differed by more than 0.5 logits. In addition, a correlation between the two sets of estimates was very high (intraclass correlation coefficient = .998). Overall, the results indicate that the DIF identified was unimportant.

## Discussion

The study shows that it is feasible to use the Rasch model to assess measurement equivalence of the CAMPHOR Activity Limitation scale. The study has provided a clear methodological framework for using the Rasch model in this process. A three-step approach was used where fit to the Rasch model was first established, measurement equivalence was assessed using DIF analyses and the importance of the identified DIF investigated.

Adequate fit to the Rasch model was achieved in the first step. Overall and item level fit statistics showed the scale had good fit to the Rasch model. Results also showed support for the functioning of the response format. Problems with the functioning of the response options have been identified frequently when Rasch analysis is applied to existing scales [29–31]. The method of responding to items differs between modes of administration, underlining the importance of assessing response option functioning. In the present study, the functioning of the response options was good with no disordered response thresholds identified. This is likely to be due to the response options for the CAMPHOR Activity Limitation scale being carefully designed using Rasch-based evidence.

Only minor evidence of DIF by type of administration was found. This was investigated in more detailed using a systematic approach outlined previously by Tennant and Pallant [28]. This determined whether the minor DIF identified had an important influence on estimates generated from the scale. This step is essential when DIF is identified in order to assess the degree of bias that items produce at the test level. The fact that two paper and pen versions were available allowed the electronic format to be compared carefully in relation to normal sample to sample variation. The results suggested that the electronic format did not differ more than the two paper and pen formats differed from each other. The results of these analyses supported the measurement equivalence of electronic and pen and paper versions of the CAMPHOR scales.

The success of the equivalence testing in this study should be considered in relation to the extent of changes in format that were made to the electronic version of the questionnaire. The new format was designed to resemble the original measure as closely as possible, having the same layout and with only minor necessary changes to the instructions. More extensive changes could well affect the Rasch fit statistics.

Two previous studies were identified that have applied IRT to assess measurement equivalence [15, 16]. One of these studies applied Rasch analysis [16]. The results of this latter study were limited to an analysis of DIF by mode of administration and no formal investigation of the impact of the DIF on overall scores was performed. The present study has provided a more detailed approach to assessing equivalence using Rasch analysis and provides a foundation for future studies.

The use of Rasch analysis to test equivalence is likely to increase in the future as it has a number of advantages over CTT approaches. Its use gives considerably more detailed information on the measurement properties of scales allowing true ‘measurement’ equivalence to be determined. Analyses based only on association or comparisons of means have limited ability to detect lack of equivalence. The study design is simpler than randomized cross over designs as only one sample for each format is required. Although two paper and pen versions were used in the present study, this would not be necessary in standard equivalence testing. There is also no need to match samples in terms of disease severity or location at which they were completed.

A potential limitation of the Rasch approach is that relatively large sample sizes are needed (approximately 150) for each format. However, similar sample sizes would provide more accurate estimates when applying CTT approaches and it is crucial to know whether different formats are equivalent in many situations. A major limitation of this approach is that relatively few PRO measures fit the Rasch model due to their age and/or the lack of a coherent measurement model. This is actually a weakness of the PRO measures as in such cases it will remain difficult to establish the equivalence of different formats of the measures.

## Conclusions

Differences between the performance of the electronic and pen and paper administrations of the CAMPHOR were minor. The results showed how the Rasch model can be used to determine the equivalence of alternative formats of PRO measures.
